# Enhancing therapeutic anti-cancer responses by combining immune checkpoint and tyrosine kinase inhibition

**DOI:** 10.1186/s12943-022-01656-z

**Published:** 2022-09-29

**Authors:** Roger J. Daly, Andrew M. Scott, Oliver Klein, Matthias Ernst

**Affiliations:** 1 Cancer Program, Monash Biomedicine Discovery Institute, Monash University, 23 Innovation Walk, Clayton, VIC 3800 Australia; 2 Department of Biochemistry & Molecular Biology, Monash University, 23 Innovation Walk, Clayton, VIC 3800 Australia; 3 Olivia Newton-John Cancer Research Institute and La Trobe University School of Cancer Medicine, 145 Studley Rd, Melbourne-Heidelberg, VIC 3084 Australia; 4 Department of Molecular Imaging & Therapy, Austin Health, and Faculty of Medicine, University of Melbourne, 145 Studley Rd, Melbourne-Heidelberg, VIC 3084 Australia

**Keywords:** Immuno-oncology, Tumor microenvironment, Targeted therapy, PD-1, PD-L1, CTLA-4

## Abstract

Over the past decade, immune checkpoint inhibitor (ICI) therapy has been established as the standard of care for many types of cancer, but the strategies employed have continued to evolve. Recently, much clinical focus has been on combining targeted therapies with ICI for the purpose of manipulating the immune setpoint. The latter concept describes the equilibrium between factors that promote and those that suppress anti-cancer immunity. Besides tumor mutational load and other cancer cell-intrinsic determinants, the immune setpoint is also governed by the cells of the tumor microenvironment and how they are coerced by cancer cells to support the survival and growth of the tumor. These regulatory mechanisms provide therapeutic opportunities to intervene and reduce immune suppression via application of small molecule inhibitors and antibody-based therapies against (receptor) tyrosine kinases and thereby improve the response to ICIs. This article reviews how tyrosine kinase signaling in the tumor microenvironment can promote immune suppression and highlights how therapeutic strategies directed against specific tyrosine kinases can be used to lower the immune setpoint and elicit more effective anti-tumor immunity.

## Introduction

Quantum leaps in cancer therapy are rare, while incremental progress remains the norm. The establishment of immune checkpoint inhibitors (ICIs) as standard of care for a growing number of cancers certainly represents a quantum leap, since they not only confer durable treatment responses, but in some cases provide a strategy to eradicate advanced human cancer. While these observations established the power of the immune system to identify and reliably kill tumor cells with a precision and durability that targeted therapies lacked, it has since become evident that the success of ICI in melanoma and certain other cancers does not apply to the majority of solid malignancies. Current efforts therefore aim to better understand the cellular and molecular mechanisms by which the anti-tumor immune response is triggered, maintained, and balanced to benefit the host without causing detrimental responses. At the same time, we can build upon our understanding of intercellular communication in the tumor microenvironment (TME) to develop strategies where ICI is combined with approved targeted therapies to confer and improve durable anti-cancer immune responses. Here we review rationalized frameworks that help to understand anti-tumor immune responses and where intersections with targeted therapies applied to the TME can bring therapeutic benefits.

### The immune-set point

Immune responses against cancer and infection share many commonalities, and therefore much of our mechanistic insight regarding optimizing effective anti-cancer immune responses has been borrowed from our understanding of the innate and adaptive immune response towards microorganisms. In common with the host’s need to balance its dealings with infections, the biggest challenge posed by ICIs is the prevention of collateral damage to tissue and concurring homeostatic processes. Indeed, a majority of clinically-approved ICIs target checkpoints that are part of negative regulatory feedback mechanisms and re-set the threshold (i.e. the *cancer-immune set point*) that has to be overcome in order to generate effective anti-cancer immunity [1]. Assuming that the cancer–immune set point is predetermined by genetics, the collective activity of the various individual regulatory immune check-points and environmental factors, this concept results in two important considerations. First, different organs or sites may have contrasting cancer–immune set points determined by the inherent immunogenicity of the respective tumor, and by the responsiveness of the individual’s immune system at that site. Second, immune-set points will be different between individuals. Determinants of set points therefore comprise the genetics of a given tumor as well as that of the patient, and most likely the extent to which anti-tumor immunity had developed prior to clinical presentation. An important corollary to this conceptual framework is the assumption that an ICI-elicited anti-cancer immune response must overcome the various immune set points encountered along the cancer immune cycle.

### The cancer immune cycle

Cancers are characterized by sequential accumulation of genetic alterations alongside the loss of normal cellular regulatory processes [2]. Inevitably, these events lead to expression of neoantigens and other cancer antigens, resulting in presentation of corresponding peptides by major histocompatibility class I (MHC-I) molecules on the surface of cancer cells. The peptide/MHC-I complexes distinguish cancer cells from their non-transformed counterparts and enables recognition by CD8+ T cells. Furthermore, an ongoing immune editing process results in continued deletion of those cancer cells that express targets for CD8+ T cells, enabling cancers to avoid elimination and suggesting a constant Darwinian co-evolution of the various immune cells during cancer progression.

**Fig. 1:**
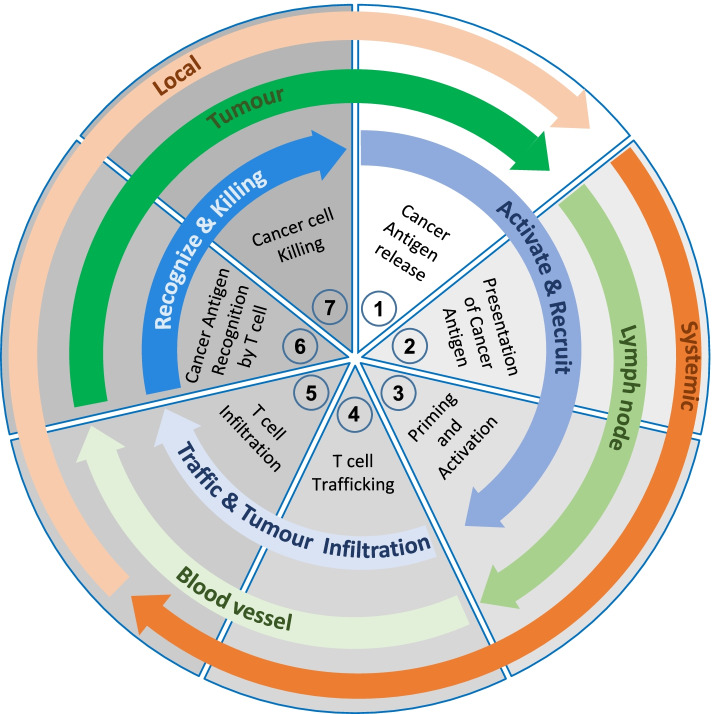
The Immune Cycle. The generation of effective anti-tumor immunity is depicted as a self-propagating, cyclic process that underpins amplification and broadening of T cell responses against specific tumor antigens. The immune cycle comprises functionally complementary stages that occur both at the site of tumor antigen release and systemically in draining lymph nodes. The three major phases include Activation and Recruitment of antigen presenting cells (stage 1-3), trafficking and tumor infiltration of T effector cells (stage 4-5) and recognition and killing of tumor cells (Stage 6-7); each phase comprises distinct functional stages that are regulated by intricate networks of positive and negative regulators (for more details refer to [1])

In order for the host to produce a productive anti-tumor immune response, a series of stepwise events must take place [1] (Fig. 1). In the first step, tumor-specific antigens that are derived from either newly expressed proteins or altered peptide processing by the cancer proteasome, are captured by dendritic cells (DCs) for processing. This process requires pro-inflammatory cytokines and/or products elicited by non-sterile cell death and possibly even microbes in the TME through pattern recognition receptor [3], to elicit antigen-specific immunity without induction of peripheral tolerance. DCs and other antigen-presenting cells then present captured antigens on MHC-I and MHC-II molecules to T cells in draining lymph nodes to prime and activate effector T cells. In the next step, activated tumor-specific effector T cells traffic to the tumor bed and infiltrate the tumor per se, where they specifically bind to the cognate antigen bound to MHCI, triggering a cascade of events that culminates in the killing of the cancer cell [4] (Fig. 1). This process will release additional tumor-associated antigens to self-sustain and amplify the extent of the immune response in subsequent rounds of these cycles.

**Fig. 2:**
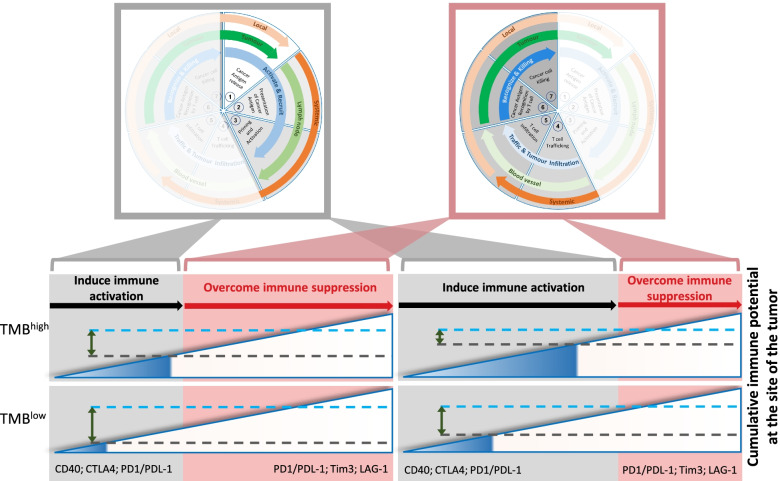
The Immune Incline. The generation of an effective anti-tumor immune response conceptionally requires overcoming of two additive barriers, namely sufficient immunogenicity to prime a maximal immune response (grey block; comprising stages 1-3 of the immune cycle, Fig. 1), and overcoming the immune suppressive activity at the site of the tumor (red block; comprising stages 4-7 of the immune cycle) that restrict activity of primed, tumor-specific effector cells. While the relative contribution of these barriers may vary between different malignancies and patients, these two barriers comprise a conceptual immune incline (wedge) with a threshold that needs to be reached for ICI to provide clinical benefits (blue broken line; set at an arbitrary and hypothetical level to illustrate the concept). For instance, tumors with higher TMB and corresponding immunogenicity have a higher *intrinsic* immune activation potential (extent of dark blue part of wedge) than tumors with lower TMB. Conversely, a strongly immune-suppressive TME (left) contributes more to the immune incline than a weakly immune-suppressive TME (right). Accordingly, ICI therapy needs to overcome the difference between the cancer cell intrinsic immune potential (black broken line) and the local immune potential required to reach the threshold for a therapeutic response (indicated by double-headed green arrows). The latter, clinically relevant ICI targets include, (1) effective antigen presentation by DC as part of inducing maximal immune activation (width of grey box) and promoted by stimulation of CD40 and/or inhibition of the CTLA4 and PD1 axis, and (2) maximal inhibition of local immune suppression in the TME (width of red box) by inhibition of the PD1 axis, Tim3, LAG1 and other inhibitor immune checkpoints

Because every stage of the cancer immune cycle builds on the same mechanisms by which the adaptive arm of the immune system defends the hosts without causing collateral damage against “self”, every step of the cycle is delicately balanced by positive and negative signals. Building on a detailed understanding of the underlying molecular mechanisms affords opportunities to enhance the immune response against cancer cells and provides the basis for the development of ICI or corresponding positive triggers of immunity. While the successful completion of the cancer immune cycle leads to an anti-tumor immune response, this is impaired in the majority of cancer patients as each step of the cycle comprises a multitude of very specific protein and cellular interactions that require tight regulation and can be negatively modulated [5]. Thus, the clinical challenge is to identify the rate-limiting steps of the cancer immune cycle in any given patient, however, insufficient immune activation and excess immune suppression remain the major two hurdles to an effective ICI-response. The extent of immunogenicity of the tumor provides the first part along a hypothetical “*immune incline*”. This provides a useful model to conceptually map the magnitude by which the systemic immune response needs to be enhanced to reach the threshold for clinical response [6] (Fig. 2). Accordingly, tumor immunogenicity is the collective outcome of the first three steps of the cancer immune cycle comprising presentation of cancer antigen(s) followed by priming and activation of a T cell response. Meanwhile, the latter part of the immune incline encompasses the last four steps of the cancer immune cycle, which provide various barriers for primed T cells to kill the cancer cells. To achieve the threshold for achieving a clinically relevant anti-tumor immune response, ICI treatment must therefore “push” tumors along the immune incline to an extent that will vary between different patients and malignancies. For instance, tumors with low immunogenicity in a tumor-suppressive TME harbour a relatively lower immune activation potential and therefore are likely to require ICI combination therapies to reach the threshold for response. By contrast, tumors with high tumor mutational burden (TMB) and which arise in an immune permissive TME present with a higher immune activation potential and are therefore more likely to respond to single agent ICI therapies [7]. Accordingly, the immune incline concept maps immuno editing [8], the process whereby oligo-clonal tumors evolve by deletion of the most antigenic clones, as a temporal reduction of the immune activation potential.

### Immune checkpoint inhibitors

To date the ICIs approved by the FDA include blocking antibodies targeting cytotoxic T-lymphocyte protein 4 (CTLA-4) (ipilimumab) and programmed cell death protein 1 (PD-1) (pembrolizumab, nivolumab, cemiplimab, dostarlimab) and its ligand PD-L1 (atezolizumab, avelumab, durvalumab) [9]. However, several other antibody ICIs have been approved by other agencies eg the anti-PD-1 antibodies sintilimab, camrelizumab and zimberelimab [10] (https://clinicaltrials.gov). CTLA-4 limits T-cell activation by competing with the co-stimulatory molecule CD28 for binding to shared ligands CD80/86 (also referred to as B7.1/B7.2). CTLA-4 is constitutively expressed on regulatory T (Treg) and effector T cells following their activation and primarily limits early T cell responses in lymphoid tissues. Meanwhile the cell-surface receptor PD-1, which binds to two ligands PD-L1 and PD-L2, is not expressed on T cells during their priming and expansion phase, but only in response to engagement of the T cell receptor. PD-L1 is expressed constitutively on many cell types including tumor cells and is induced in immune cells following their exposure to IFN-γ and other cytokines. Meanwhile, PD-L2 expression is mainly limited to activated DCs. Engagement of either ligand results in the ‘exhausted’ phenotype of effector T cells characterized by reduced T cell proliferation, glucose metabolism and cytokine production alongside shortened T cell survival.

Immunotherapy using monoclonal antibodies blocking PD-1 or PD-L1 suggest no major differences in efficacy and toxicity between the two treatment modalities as monotherapies, at least in non-small cell lung and bladder cancer [11]. While this may be expected from inhibition of a cognate ligand/receptor pair, the underlying immunological mechanisms partially differ in response to anti-PD-1 and anti-PD-L1 therapy, not least because of the differential requirement of the respective antibodies for FcγR engagement to achieve in vivo efficacy [12]. Indeed, anti-PD-L1 treatment has been associated with less severe adverse events [13], suggesting that the differences between anti-PD1 and anti-PD-L1 agents could be clinically exploited for better tailoring of treatments to the tumor characteristics of an individual patient.

Immunotherapy using anti-PD-1 blockade has demonstrated significant clinical activity across a range of malignancies and anti-PD-1/CTLA-4 dual blockade has shown superiority compared to the corresponding monotherapies in melanoma, MSI-high colorectal and renal cell cancer [9]. The current paradigm states that anti-PD-1 blockade mediates its therapeutic effect by reinvigoration of tumor specific effector cells in the TME in response to high affinity neo-antigens. By contrast, CTLA-4 blockade facilitates priming of naïve tumor specific T cells or reactivation of memory cells by DCs in secondary lymphoid organs [14]. However, emerging pre-clinical studies demonstrate that a sustained anti-tumor immune response induced by PD-1 blockade may rely on the influx of new T cell clones into the tumor [15]. One mechanism that could account for the latter observation is that simultaneous blockade of both checkpoints occurs during the priming and/or reactivation stage of tumor specific T cells in secondary lymphoid organs, where PD1/PD-L1 signaling restrains immunity in lymph nodes [16]. Indeed, combined anti-CTLA-4 and anti-PD-1 blockade can overcome the larger immune incline presented by less immunogenic cancers and associated poorer T cell activation resulting from exposure to low affinity antigens (Fig. 2) [17]. This conclusion is supported by two converging observations. First, TMB, as a surrogate measure for tumor immunogenicity, predicts responses less reliably in patients treated with combined anti-CTLA-4/PD-1 blockade than with anti-PD-1 monotherapy [18, 19]. Second, the frequency of severe autoimmune related toxicities is higher in patients receiving dual checkpoint blockade than those treated with anti-PD-1 monotherapy [18, 19].

Additional immune checkpoint regulators with emerging clinical relevance include lymphocyte-activation gene 3 (LAG-3/CD223) expressed on activated T cells, NK cells and plasmacytoid DCs, and TIGIT expressed on effector T and NK cells, which binds to CD155 on antigen-presenting cells or tumor cells. Thus, concomitant anti-LAG-3 blockade with anti-PD-1 treatment provides superior outcomes than anti-PD-1 monotherapy in patients with metastatic melanoma [20]. Significantly, combining the latter treatments reduces the extent of severe immune related toxicity when compared to combined anti-CTLA-4/PD-1 therapy. Indeed, toxicity associated with anti-PD-1/LAG-3 combination therapy is only slightly higher than with anti-PD-1 monotherapy. This suggests that dual PD-1/LAG-3 blockade interferes to a lesser degree with the overall immune homeostasis and may preferentially target T cells in the TME [20]. While immune checkpoints that are currently targeted therapeutically by monoclonal antibodies are surface molecules that act as negative regulators on lymphoid effector cells, there is emerging evidence in pre-clinical models that intracellular checkpoints, such as cytokine-inducible SH2-containing protein (CISH), the E3 ubiquitin ligase Cbl-b, and protein tyrosine phosphatase PTP-1B are also of importance and represent potential therapeutic targets [21–24].

### Tumor immune phenotypes

**Fig. 3:**
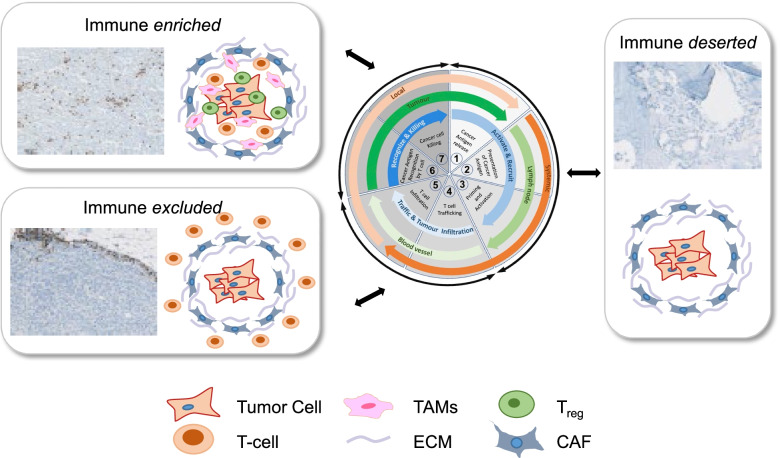
Immunophenotypes and their relation to immune cycle dysfunction. Based on distribution of effector T cells, revealed by staining for anti-CD3 reactive cells, tumors can be classified into three immunophenotypes. *Immune-deserted tumors* show an absence of effector T cells due to a lack of immunogenicity leading to poor T cell activation (stages 1-3 of the immune cycle, centre). *Immune-excluded tumors* show T cell accumulation surrounding the tumor parenchyma resulting from impaired trafficking and tumor infiltration (stages 4-5). *Immune-enriched tumors* show infiltration with functionally impaired T cells as a result of immune suppressive activities conferred by Tregs and other negative regulatory cells (stages 6-7)

Broadly, human tumors can be separated according to the distribution of immune cells in the tumor parenchyma, the invasive margin and the tumor core to yield three cancer immune phenotypes [25, 26] (Fig. 3).

*Immune-deserted tumors* lack the presence of CD8-expressing effector cells both in the tumor and parenchyma, reflecting either immunological ignorance, the presence of immune tolerance, or a lack of appropriate T-cell priming and activation [25, 27–29]. While the presence of myeloid and other suppressive cells may vary, these tumors rarely respond to anti-PD-L1/PD-1 therapy [29]. This phenotype occurs often in brain, thyroid, pancreatic and prostate cancers [30], and has been associated with an absence of pre-existing anti-tumor immunity that correlates with low TMB. Consistent with an assumption that the rate-limiting step for immune-deserted tumors is the generation of tumor-specific T cells, non-small cell lung cancers exhibiting inactivating mutations in LKB1 exhibit a poor response to anti-PD1 immunotherapy, despite a high TMB, due to impaired antigen presentation resulting from reduced expression of the immunoproteasome. However, a lower proteasome activity can result in enhanced autophagy as a compensatory mechanism. Reflecting the inter-relationship of the proteasome and autophagy pathways, inhibition of autophagy by targeting ULK1 restored antigen presentation and synergized with PD-1 antibody blockade to promote tumor regression in Lkb1-mutant mice. Furthermore, ICI response could be improved by treatment with chloroquine [31], because inhibition of autophagy restored immunoproteasome activity and antigen presentation, and autophagy is required to resist the cytotoxic activities of IFNγ and TNF [32].

Other therapeutic interventions applied in immune desert tumors aim for a complimentary increase of tumor antigenicity. For instance, radiotherapy has been identified as a treatment modality that enables immunogenic cell death via activation of the STING pathway, and an abscopal effect has been observed as a rare clinical phenomenon in cancer patients receiving radiotherapy [33]. Although there are numerous current trials exploring the combination of PD-1/PD-L1-based ICI therapy with radiotherapy, emerging data suggest a more beneficial effect when radiotherapy is combined with CTLA-4 rather than PD-1/PD-L1 based ICI therapy [34, 35]. It remains to be established whether this relates to the different mechanisms of action of the two types of ICI, or differences between the tumor types studied.

*Immune-excluded tumors* show an abundant presence of effector T cells within the stroma surrounding tumor cells but not penetrating the tumor parenchyme [29, 36, 37]. The immune-excluded phenotype is associated with expression of CCL2, IL6, IL10, TGFβ, and other chemokine/cytokines as well as with cell types associated with immune suppression or tolerance. Meanwhile, effector T cells are accompanied by Treg at inflammatory sites to maintain immune homeostasis, even in the presence of an active anti-tumor immune response [38, 39]. Immune-excluded tumors can also result from the presence of vascular barriers or immune suppressive cancer associated fibroblasts (CAFs), or CAF-mediated excessive deposition of extracellular matrix and desmoplastic encapsulation of tumor cells. In response to ICI treatment, stroma-associated T cells can undergo activation and proliferation, but not infiltration and therefore clinical responses remain rare in immune-excluded tumors. The corollary to this is the presence of a pre-existing anti-tumor response that is rendered ineffective by retention of immune cells in the stroma. Emerging insights suggest that inhibition of TGFβ signalling and/or the use of anti-angiogenic agents (see below) may show therapeutic benefits in this tumor immune phenotype [40].

*Immune-enriched tumors*, also referred to inflamed tumors, have a parenchyma characterized by the presence of various immune cell types, including CD4- and CD8-expressing effector T cells, inhibitory Tregs, myeloid-derived suppressor cells, suppressor B cells and CAFs. These cells are positioned in proximity to the tumor cells, and the CD8 cells often demonstrate an exhausted, dysfunctional state [29, 41, 42]. Meanwhile, tumor cells can show downregulation of MHC-I expression and other pathways that protect from immune detection. Immune-enriched tumors may exhibit staining for PD-L1 on infiltrating immune and tumor cells [29, 41], and show abundance of type I and II IFN, IL12, IL23, IL1β, TNF, IL2, granzymes, CXCL9 and CXCL10 and other pro-inflammatory/effector cytokines [29, 41, 42]. These characteristics suggests a pre-existing anti-tumor immune response that is rendered inactive by tumor intrinsic and extrinsic factors. Indeed, clinical responses to anti-PD-L1/PD-1 therapy occur most often in patients with inflamed tumors [29, 41, 42].

While this histological classification is useful, more refined classification based on molecular signatures is now being pursued, as transcriptomic analysis at the level of individual cells and characterization of their spatial location is becoming more accessible. However, personalised immunotherapy will always focus on the most dominant immune phenotype to achieve durable tumor control, while acknowledging that the tumor immunophenotypes and TME not only remain dynamic, but also may differ between individual metastases in any given patient [43].

### Cells of the TME shape the ICI response

**Fig. 4:**
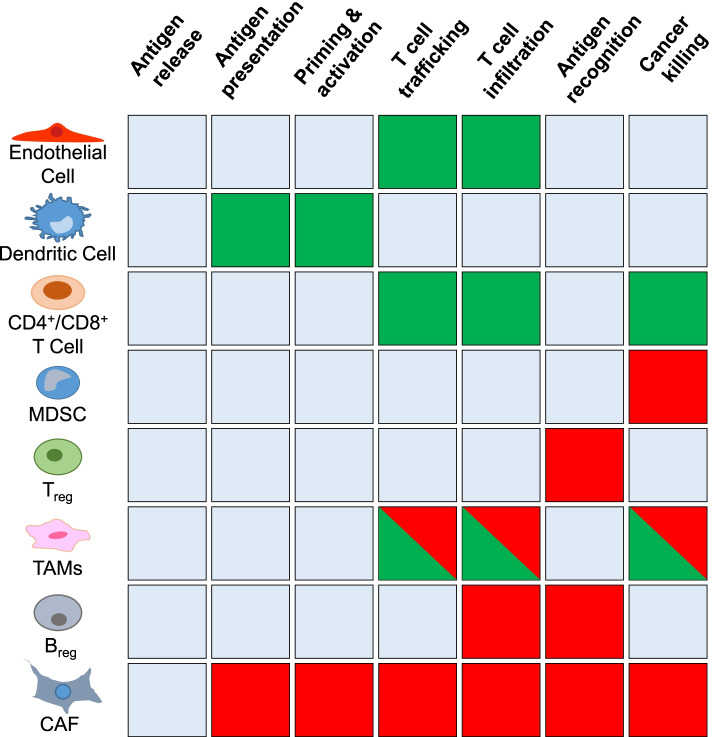
Cell types of the tumor microenvironment and their relationship to the immune cycle. Schematic depiction of the major cell types of the tumor microenvironment involved in promoting (green shading) or suppressing (red shading) the anti-tumor response of immune checkpoint inhibitor therapies, and the stages of the immune cycle that these cells impact. The dual shading for TAMs indicates the opposing functions of the two TAM endotypes. CAF, cancer-associated fibroblast; MDSC, myeloid-derived suppressor cell; TAM, tumor associated macrophage; Treg, regulatory T cell

**Fig. 5:**
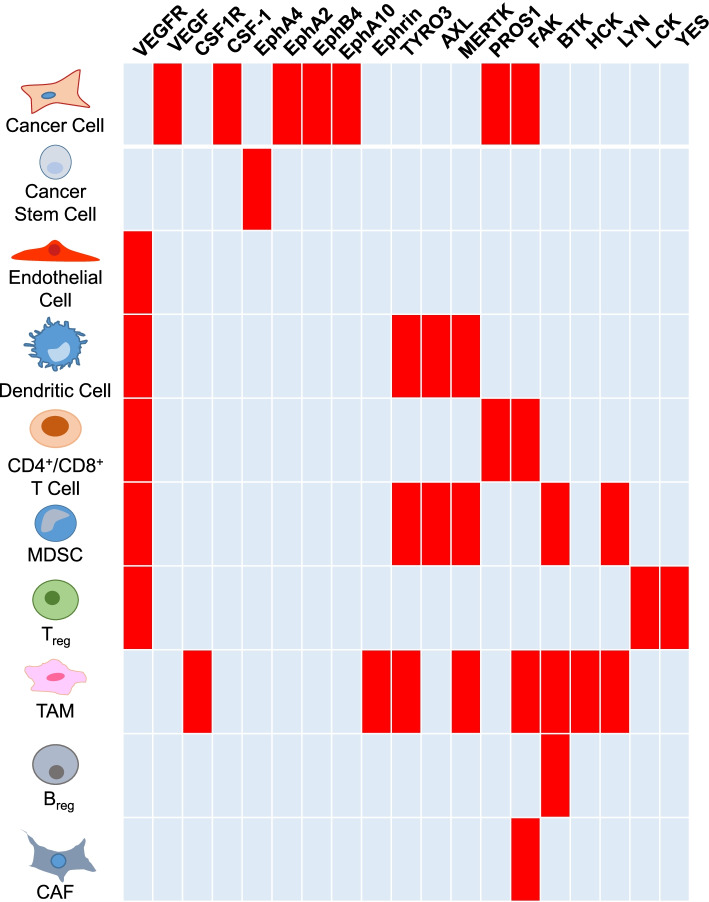
Functional profile of (Receptor) Tyrosine Kinases and their ligands across different cell types in the tumor microenvironment. Red shading indicates a cell type where a functional role of a given kinase or its ligand relevant to ICI efficacy has been demonstrated, for example by gene knock-out or pharmacological inhibition. Note that this may not indicate the expression profile per se, but where expression in a particular cell type has been linked to immunosuppression in the TME and the kinase/ligand represents a potential target for combination therapy with ICI. For details on each cell type please refer to text

The TME represents a community of various hematopoietic and non-hematopoietic cell types that are genetically more stable than tumor cells. Compelling experimental evidence confirms that cancer cells corrupt and coerce their normal counterparts in the TME to adopt tumor-promoting and immune-suppressing characteristics, many of which remain phylogenetically hardwired as part of evolutionary conserved wound-healing and tissue regeneration mechanisms [44]. Collectively, the various cell types of the TME engage in reciprocal communication involving a plethora of growth factors, cytokines and adhesion molecules that play fundamental roles in promoting tumor progression and shaping the response to ICI therapy (Fig. 4). While this field has been summarized in detail by others [45], key features of the main cell types are summarized below. Importantly, a variety of tyrosine kinases participate in intercellular communication within the TME in a cell type-selective manner and contribute to immunosuppression, establishing them as potential targets for therapies aimed at improving ICI response (Fig. 5).

*Endothelial cells* represent the fundamental building block of the tumor vasculature, which plays a key role in delivering nutrients and oxygen to the tumor. Establishment of the tumor vasculature can involve angiogenesis (formation of new blood vessels), hi-jacking of existing vessels, and trans-differentiation of cancer cells to endothelial cells in a process termed vascular mimicry [46]. While providing critical life-support to tumors, the abnormal and dysfunctional nature of the tumor vasculature contributes to tumor suppression by impairing trafficking of cytotoxic T cells into the tumor [47]. As discussed later, a key regulator of vasculo - and angiogenesis is vascular endothelial growth factor (VEGF) signalling via their cognate receptor tyrosine kinases (RTKs), the VEGFRs [46]. However this signalling axis also regulates other cell types within the TME, including dendritic cells (DCs), CD8+ T cells, myeloid-derived suppressor cells (MDSCs) and regulatory T cells (Tregs) (Fig. 5).

*Tumor-associated macrophages* (TAM) account for one of the most prominent cell types shaping an immune suppressive TME. While conventional activated, pro-inflammatory (M1-like) macrophages act as scavenger cells, their alternative activated, anti-inflammatory (M2-like) endotype promotes angiogenesis and tissue remodeling. However, the dichotomic M1/M2 classification does not accurately describe the in vivo heterogeneity of TAMs, which often have traits of both polarization states [48]. Alternative activated endotypes promote cellular proliferation and blood vessel development and through expression of PD-L1 and PD-L2 help suppress collateral damage by the immune system. Alternative activated macrophages also express less antigen-presenting MHC-II [8], indicating that reprogramming of TAMs towards M1-like endotypes can augment their role as antigen presenting cells. This reprogramming is augmented following engagement of CD40, expressed on macrophages and many other immune cells, by CD40 agonist antibodies [49, 50]. Signalling by the Colony stimulating factor-1 receptor (CSF-1R), as well as downstream Src family kinases, such as Hck, represent potential therapeutic targets in TAMs [51, 52] (Fig. 5).

*Tumor-associated neutrophils* (TAN) are often associated with poor patient prognosis and response to therapy [53]. TANs are recruited into the tumor parenchyma via monocyte chemoattractant protein 2 (MCP-2), macrophage inflammatory protein 1α/β and other cognate ligands. Meanwhile, IL6 produced by CAFs not only induces PD-L1 and TNFα expression on TANs to increase their survival and immune-suppressive activity [54], but TNFα signaling can then feed forward to stimulate IL17 secretion and further promote TAN recruitment.

*Myeloid-derived suppressor cells* (MDSCs) are immature myeloid cells defined by their immunosuppressive functions [55]. MDSCs are classified into the phenotypically distinct polymorphonuclear/granulocytic-MDSCs (PMN-MDSCs/G-MDSCs) and mononuclear-MDSCs (M-MDSCs). M-MDSCs have metabolic adaptivity to sustain their immune suppressive functions in a nutrient-depleted TME [56]. Importantly, M-MDSCs are molecularly distinct from M1−/M2-like macrophages and depend on distinct transcriptional networks that govern M-MDSC maturation from polymorphonuclear neutrophils and monocytes.

*Regulatory T cells* (Tregs) are a specialized subpopulation of CD4+ T cells that express the nuclear transcription factor FoxP3 and help maintain homeostasis and self-tolerance by suppressing immune responses [57]. Natural Tregs express CTLA-4 and can produce TGFβ, IL-10, adenosine and other messengers of immunosuppressive signalling. Abundant Treg infiltration is a common feature of tumors with poor response to ICI, and this may be antagonised with strategies that impair Treg differentiation or exploit their recognition by anti-CTLA4 antibodies.

*Tumor-associated B cells* can promote tumor inflammation [58], for instance through B cell receptor stimulation by melanoma derived antigens [59], and also inhibit T cell-dependent therapy responses through the release of IL-10 and TGF-β [60]. The functions of regulatory B cells are induced by IL-35, and in humans, their abundance commonly increases with tumor progression [61]. Indeed, B cell depletion by anti-CD20 antibodies in end-stage melanoma patients can provide objective responses [62]. However, depletion of B cells by anti-CD20 antibodies in melanoma patients decreases tumor-associated inflammation and CD8+ T cell numbers and CD138+ plasmablast-like cells increase T cell activation in response to anti-PD-1 blockade in vitro. Indeed, the frequency of plasmablast-like cells can predict ICI response in melanoma patients [63].

Dendritic cells (DC) are the most potent antigen presenting cell type, with the conventional cDC1 playing the most important role in presentation of tumor antigens despite accounting for the rarest subset of all DC. Tumor-resident cDC1s are the predominant sources of the T-cell chemokines underpinning the recruitment of effector T cells into the tumor [64]. While pre-clinical studies demonstrated improved tumor control when combining anti-PD-L1 blockade with administration of Flt3L as the main growth factor for DCs, administration of recombinant Flt3L as a monotherapy failed to show efficacy in patients with advanced malignancies. Because these observations may reflect the immature phenotype of the induced DCs and associated concomitant expansion of Tregs [65], Flt3L treatment may be more efficacious when combined with agents providing DC maturation signals such as a CD40 agonist [66] or TLR ligands [67].

*Natural killer* (NK) cells harbour constitutive ready-to-kill machinery that does not require antigen sensitization, and which can kill target cells through cytotoxic processes as well as indirectly via antibody-dependent cell-mediated cytotoxicity [68]. While NK cells provide important immune surveillance against circulating tumor cells [69], the activity of circulating NK cells generally decreases with disease progression. Indeed, similar to the negative feedback mechanisms regulating T cells, activated NK cells express PD-1, CTLA4, TIM3, LAG3, and other immune checkpoint receptors, explaining why NK cells become increasingly dysfunctional with cancer progression [70].

*Innate lymphoid cells* (ILCs) are innate counterparts of T cells that contribute to immune responses through their secretion of primarily Th2-type effector cytokines (IL5, − 9, − 13, etc) thereby regulating the functions of other innate and adaptive immune cells [71]. ILCs are present in lymphoid and non-lymphoid organs and are particularly abundant at the mucosal barriers, where they are exposed to allergens, commensal microbes, and pathogens, and play an important role in coordinating the wound-healing response via IL-17, − 25 and − 33, thus being implicated in promoting tumor development.

*Cancer-associated fibroblasts* (CAFs) are master regulators of the fibrotic responses within the TME, and also play pivotal roles during tumor growth and metastasis, angiogenesis and immune suppression. They either display secretory inflammatory (iCAF) phenotypes, or myofibroblastic (myCAF) phenotypes characterized by extracellular matrix production [72]. myCAFs surround tumor ducts and interact with tumor cells through juxtacrine mechanisms, while iCAFs reside at greater distances within the stroma and interact via secretion of inflammatory cytokines with tumor cells, myCAFs and other stromal cells. Recent studies suggest additional heterogeneity of CAFs [73] reflecting diverse origins including from epithelial cells, tissue-resident stellate cells, adipose tissue, and the bone marrow, and include MHCII-expressing CAFs able to stimulate proliferation of CD4+ T cells *ex vivo* [74].

Fibroblast activation protein (FAP)-expressing CAFs mediate tumor-promoting immunosuppression via recruitment of MDSCs [75]. Immune suppression by FAP^+^ CAFs is mediated by CXCL12 via the CXCR4 receptor, and inhibition of CXCR4 led to the elimination of cancer cells by CD8^+^ T cells [76]. FAP^+^ fibroblasts exhibit upregulated PTEN/Akt and MEK/Erk signalling, secrete CCL2, IL-6 and CXCL8, and induce migration of esophageal squamous carcinoma cells and M2 polarization of macrophage-like cells [77]. FAP+ CAFs also promote accumulation of CD4^+^CD25^+^ T cells and enhance their differentiation to Tregs [73]. Overall, the different CAF subtypes elicit broad immunosuppressive effects in the TME, reflecting their interaction with diverse cell types including TAMs, DCs, Tregs, cytotoxic T cells and MDSCs [78] (Fig. 4).

### Combining tyrosine kinase and immune checkpoint inhibition

Given the complexity of cell types within the TME, therapeutic disruption of the complex intercellular dialogue appears a challenging task. However, a number of tyrosine kinases have recently emerged as key components of immunosuppressive signalling networks in the TME, highlighting exciting opportunities for drug development including the re-purposing of existing drugs. As the majority of these kinases promote the immune suppressive TME, their inhibition presents opportunities to reduce the immune incline and to elicit therapeutic responses. A summary of the most prominent tyrosine kinases expressed across specific cell types within the TME and linked to immunosuppression are highlighted in Fig. 5. It should be noted that the majority of clinical developments at this stage focus on inhibition of kinases that impede specific aspects of the cancer immune cycle. However, other components of tyrosine kinase signalling cascades also represent therapeutic targets, such as the protein tyrosine phosphatase PTP1B, which is increased in intra-tumoral T cells to limit T cell expansion and cytotoxicity [24].

#### Receptor tyrosine kinases

##### Vascular endothelial growth factor receptors (VEGFRs)

**Fig. 6:**
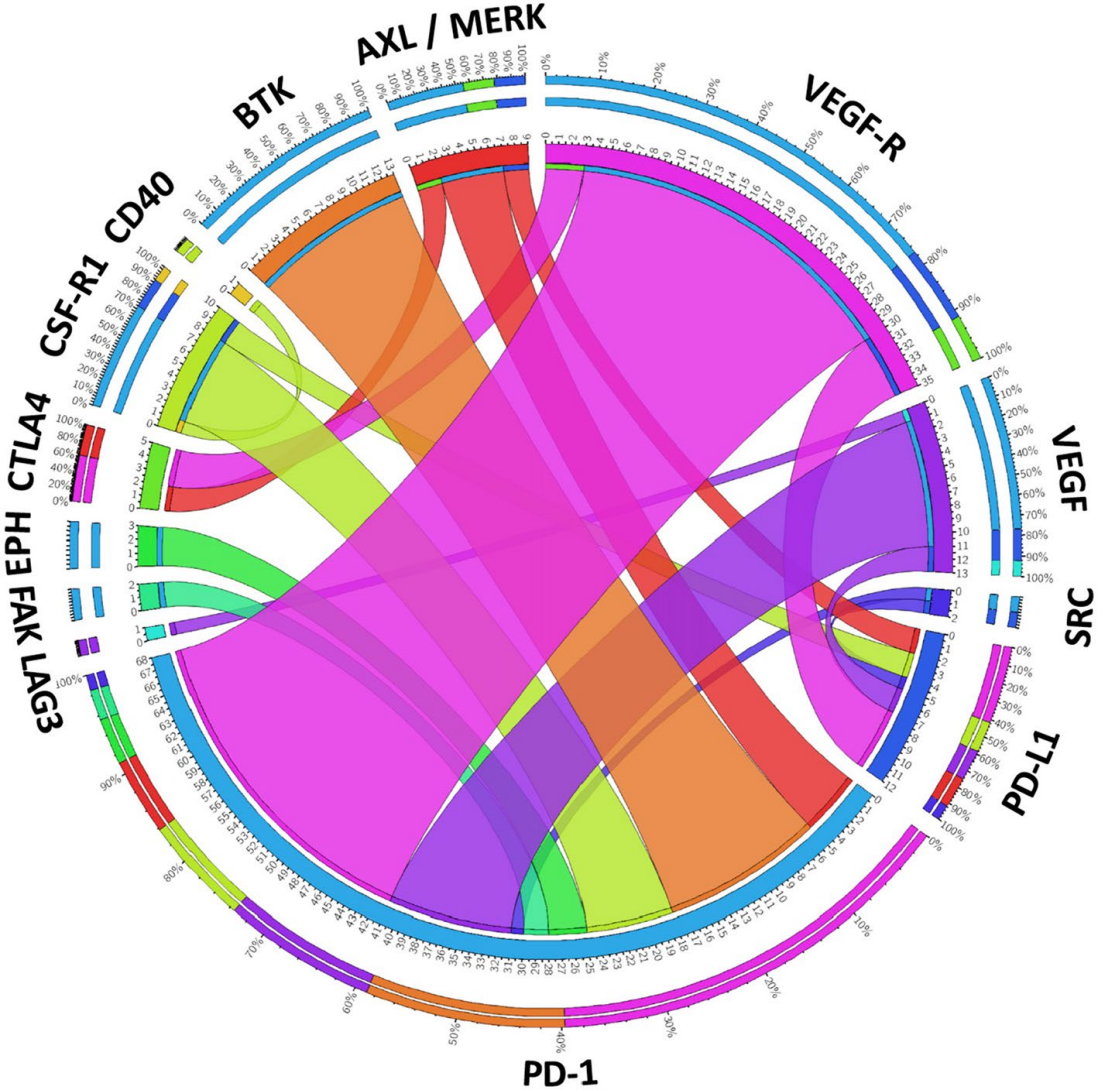
Current combinations of ICI with targeted therapies directed against tyrosine kinase pathways. Circos plot visualising the frequency of combining specific ICI with inhibition of (receptor) tyrosine kinase signaling pathways in current clinical trials (as listed on https://clinicaltrials.gov). The width of the bands is proportional to the number of corresponding combinations. The absolute number of trials for each target is shown on the inner ring, and the relative distribution across all combinations for a specific target is depicted on the outer ring. For information on the specific malignancies included in these trials, refer to Table 1

Vascular endothelial growth factor (VEGF) belongs to a family of growth factors that includes VEGF(A-D) and placental growth factor [79]. The receptor tyrosine kinases (RTKs), VEGFR1-3 exhibit selective binding patterns to the aforementioned ligands [79], with signalling via VEGFR2 being the predominant signalling mechanism regulating endothelial cell biology, vascular permeability, alongside vasculo- and angiogenesis [79]. The main mediator of tumor angiogenesis is VEGF-A, usually referred to as VEGF, which is overexpressed by cancer cells and CAFs of many human tumors, in response to hypoxia [80, 81] and other triggers. While VEGF production promotes angiogenesis, in cancers these new blood vessels are often abnormal and leaky [80]. Given the importance of angiogenesis to cancer development and progression, a variety of strategies have yielded FDA-approved therapies to block VEGF signalling. Most notably, they include the anti-VEGF-A monoclonal antibody Bevacizumab (approved for colorectal, metastatic non-small cell lung, renal cell, and other cancers), and the decoy receptor ziv-Aflibercept (approved for metastatic colorectal cancer). These biologicals are complemented by tyrosine kinase inhibitors (TKIs) such as Axitinib, Lenvatinib, Vandetanib, Sorafenib and Sunitinib that all target VEGFRs among other kinases. These TKIs are used for treatment of several cancers depending on the TKI including renal cell and hepatocellular carcinoma and thyroid cancer [80] (Fig. 6; Table 1).

**Table 1:**
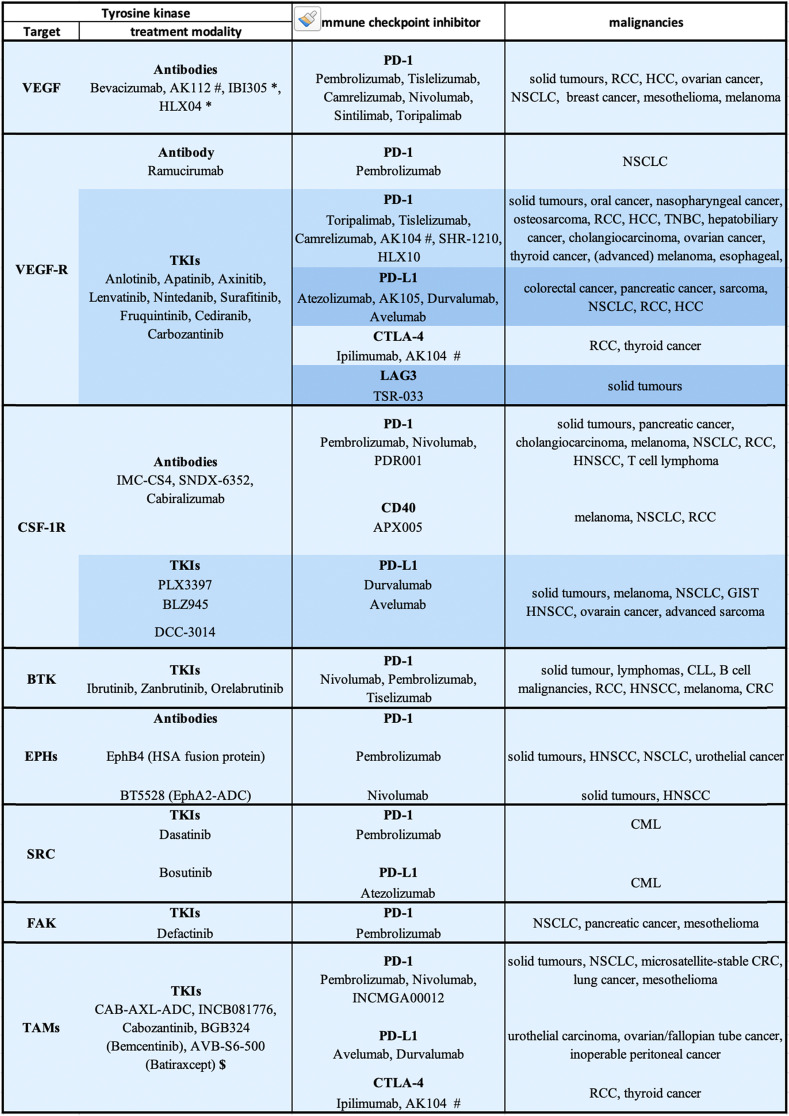
Active clinical trials of TKI therapies and immune checkpoint inhibitors in cancer patients

*Abbreviations*: *CML* chronic myeloid leukemia, *CRC* colorectal cancer, *GIST* gastrointestinal stromal tumor, *HCC* hepatocellular carcinoma, *HNSCC* head and neck squamous cell carcinoma, *NSCLC* non-small cell lung cancer, *RCC* renal cell carcinoma, *TNBC* triple negative breast cancer

* Biosimilar of Bevacizumab

# bispecific antibody also binding to PD1

$ Extracellular domain trap

It is now evident that inhibition of VEGF function in cancer can lower immunosuppression and improve the efficacy of ICI therapy, with this reflecting the wide range of biological actions of VEGF. One such activity is promotion of an abnormal tumor vasculature. Here, use of a low, vascular-normalizing dose of anti-VEGFR2 antibody led to increased tumor infiltration of CD4+ and CD8+ T cells and improved the efficacy of a cancer vaccine therapy in a CD8+ T cell-dependent manner in mouse breast cancer models [47]. In addition, VEGF decreases the expression of specific adhesion molecules on the tumor vascular endothelium required for efficient trafficking of T cells into the tumor [82]. Likewise, VEGF in combination with IL10 and prostaglandin E2 (PGE2) promotes a tumor endothelial ‘death barrier’ that is established by Fas-L expression to exclude CD8+ T cells preferentially over Tregs [83]. VEGF also prevents the maturation of DCs and hence antigen presentation [84]. In mature DCs, VEGF promotes PD-L1 expression [85] and suppresses their capacity to activate T cells [86]. Meanwhile in CD8+ T cells, VEGF induces expression of PD-1, CTLA-4, Tim-3 [87] and other inhibitory receptors associated with T cell exhaustion. Consistent with this observation, combination treatment of a high VEGF-expressing mouse model of colon cancer with antibodies against VEGF and PD-1 was significantly more effective than either monotherapy [87].

In addition to its effects on DCs and CD8+ T cells, VEGF also regulates the function of immunosuppressive cells in the TME by promoting expansion of MDSCs [88]. This effect could be reversed in colon and renal cell tumor models following treatment with Bevacizumab and/or sunitinib [89, 90]. Furthermore, VEGF directly promotes the proliferation of Tregs via VEGFR2, and in a mouse model of colon cancer, administration of anti-VEGF antibody or sunitinib decreased Tregs. Furthermore, Bevacizumab inhibited Treg accumulation in peripheral blood of patients with metastatic colon cancer [91].

Characterization of the regulatory mechanisms described above has led to intensive pre-clinical and clinical testing of combination strategies involving targeting of the VEGF axis and ICI (Fig. 6), culminating in FDA-approval for pembrolizumab or Avelumab and Axitinib for renal cell carcinoma [92, 93], pembrolizumab and Lenvatinib in endometrial carcinoma [94], Atezolizumab, Bevacizumab and chemotherapy in non-squamous non-small cell lung cancer [95] and Atezolizumab and Bevacizumab in unresectable hepatocellular carcinoma [96].

##### Colony stimulating factor-1 receptor (CSF-1R)

CSF-1R is a transmembrane receptor tyrosine kinase that acts as a receptor for both colony stimulating factor-1 (CSF-1) and IL34 [97]. The CSF-1R is of major interest in the context of immunosuppressive signalling in the TME, because of its fundamental role in regulating differentiation, proliferation, survival and migration of TAMs and other myeloid cells [51]. TAMs impair mobilization of effector T cells and their activation through expression of inhibitory checkpoint molecules (e.g. PD-L1), uptake of immunotherapeutic antibodies via FcγR binding, secretion of immunosuppressive cytokines (e.g. TGFβ), recruitment of Treg (e.g. via production of the chemokine CCL20) and induction of extracellular matrix remodelling [51, 98]. Therapeutic targeting of CSF-1R signalling is achieved via use of monoclonal antibodies that target CSF-1 (e.g., MCS110, PD-0360324) or the extracellular region of the receptor (e.g. LY3022855, SNDX-6352, Cabiralizumab, IMV-CS4), or TKIs that exhibit selective inhibitory activity for the CSF-1R kinase (e.g. PLX-3397/Pexidartinib, BLZ945, ARRY-382, DCC-3014). Encouraging pre-clinical data support the use of these approaches to enhance the efficacy of ICI therapy. For example, treatment of a mouse model of pancreatic ductal adenocarcinoma (PDAC) with Pexidartinib suppressed TAM accumulation and M2-like polarization thereby enabling better DC-dependent antigen presentation and enhanced T cell activation. In addition, combining CSF-1R inhibitors with either anti-PD1 or anti-CTLA4 blockade markedly enhanced efficacy when compared to ICI monotherapy [99]. Likewise, co-treatment of mice bearing BRAF V600E-driven melanoma with CSF-1R- and PD1-blocking antibodies increased efficacy when compared to single agent treatments [100], most likely due to the capacity of melanoma cell-derived CSF-1 to enhance TAM recruitment.

To date, the major clinical impact for anti-CSF-1R therapy has been with Pexidartinib for treatment of tenosynovial giant cell tumors, a locally aggressive malignancy that overexpresses CSF-1. Although Pexidartinib was FDA-approved for treatment of this malignancy [101], it is associated with severe morbidity and not amenable to improvement with surgery. However, for other solid cancers, while administration of anti-CSF-1R therapies as single agents is tolerated with manageable toxicities, clinical efficacy has been either limited or lacking [102, 103]. This has led to numerous trials combining these approaches with ICI (Fig. 6; Table 1).

##### Eph receptors

The Ephs represent the largest family of RTKs, with 14 members classified into groups A and B based on homology and binding to either ephrin type A or B ligands, respectively [104]. Signalling by specific Eph/ephrin combinations plays critical roles in normal development and also in cancer, where aberrant expression and/or function of specific Ephs and ephrins characterizes particular malignant cell types and also the TME [104, 105]. Accordingly, a variety of therapeutic approaches targeting the Eph/ephrin system in cancer are in either pre-clinical development or clinical trials, including antibodies and antibody-drug conjugates, Eph-fusion proteins and multi-kinase inhibitors that exhibit Eph receptor specificity [104, 106] (Fig. 6; Table 1).

In addition to a large body of work documenting direct effects of targeting the Eph/ephrin system in malignant cells [104], increasing evidence indicates that Eph/ephrin signalling in cancer contributes to immunosuppression in the TME. For example, in breast cancer, engagement of EphA4 expressed on cancer stem cells by its cognate ligand on TAMs, triggers cytokine release by the former that promotes their stem cell state and polarizes TAMs towards tumor-promoting endotypes [107]. Meanwhile in pancreatic ductal adenocarcinoma, EphA2 expression negatively correlates with T cell infiltration, leading to discovery of an immunosuppressive EphA2/TGFβ/COX-2 signalling axis. Indeed, deletion of EphA2, or inhibition of COX-2, restored sensitivity to ICI therapy in preclinical models [108]. In contrast, in head and neck squamous cell carcinoma (HNSCC), a different Eph receptor is implicated, EphB4. Interaction of EphB4 with its ligand ephrin B2 plays critical roles in both normal development and cancer progression [109], and in HNSCC, EphB4 is highly expressed in cancer and stromal cells, with lower expression of ephrin B2 [110]. In mouse models of this malignancy, blocking EphB4/ephrin B2 interaction with a competing peptide resulted in decreased Treg infiltration and increased CD4+ and CD8+ T cell activation in tumors, together with an increase in M1 and reciprocal decrease in M2 macrophages. In addition, combining radiotherapy with this approach resulted in a similar inhibition of tumor growth as combining the former with anti-PD-L1 immunotherapy [110]. Another link between the Ephs and the immune response to tumors is provided by the regulatory relationship between EPHA10 and PD-L1. Expression of these two proteins is strongly positively correlated in breast cancer, and juxtacrine signalling by EPHA10 in breast cancer cells leads to enhanced expression of PD-L1. Indeed, EPHA10 deletion in a syngeneic mouse model of breast cancer led to decreased tumor growth and increased CD8+ T cell infiltration [111].

While targeting the Eph receptor signalling system in combination with checkpoint blockade has only begun to be explored in the clinic (Fig. 6; Table 1), EPHA7 mutations have recently emerged as a potential predictive biomarker for positive response to ICI therapy across several cancer types [112].

##### The TAM family of RTKs

This family, comprising TYRO3, AXL and MERTK, together with their ligands GAS6 and PROS1, function at the interface of innate and adaptive immunity to suppress immune responses and inflammation [113]. This is achieved via a number of mechanisms, including regulation of DC function and phagocytic clearance of apoptotic cells by macrophages and other phagocytic cells. Aberrant signalling by the TAM RTKs is characteristic of autoimmune diseases and cancers. In tumors, perturbed TAM family expression is evident in both the cancer cells themselves, where these receptors promote processes such as cell proliferation, survival and migration, and specific cell types in the TME, where in general they have immunosuppressive and anti-inflammatory roles [113, 114]. The emergence of the TAM family of RTKs as therapeutic targets has led to the development of a variety of potential strategies for their targeting. These include multi-TKIs that exhibit some selectivity for these RTKs (eg BGB324/Bemcentinib, Cabozantinib, TP-0903, MGCD-65/Glesatinib, INCB081776), monoclonal antibodies (e.g. YW327.6S2 and MAb173), antibody-drug conjugates (e.g. CAB-AXL-ADC), and decoy receptors (e.g. AVB-S6-500) [114–117] (Fig. 6; Table 1).

The TAM family are implicated in regulating MDSC function, since increased MDSC expression of TYRO3, AXL and MERTK and their ligands was detected in a syngeneic mouse model of melanoma, and circulating MERTK^+^ and TYRO3^+^ MDSCs were more abundant in patients with metastatic melanoma [115]. Indeed, engineered deficiency of either of these receptors in MDSCs conferred lower immunosuppressive functions associated with impaired migration of MDSCs to tumor-draining lymph nodes, resulting in reduced melanoma tumor formation. Consistent with these findings, pharmacological inhibition of these receptors with UNC4241 also diminished MDSC suppressor function, increased CD8^+^ T cell infiltration into tumors, and reduced tumor growth. Moreover, UNC4241 enhanced the effects of anti-PD1 therapy [115]. These RTKs also mediate immunosuppression in DCs, where production of the ligand PROS1 by activated T cells restrains DC activation and cytokine production [118, 119]. In addition, tumor-secreted PROS1 can bind to MER and TYRO3 on macrophages and suppress their ability to express an M1, inflammatory and anti-tumor phenotype [120], and binding of GAS6 and PROS1 to phosphatidylserine exposed on the outer membrane leaflet of apoptotic cells can activate TAM receptors on macrophages and stimulate uptake of the dying cells (efferocytosis), which also reduces inflammation in the TME [121]. However, while these studies support therapeutic targeting of the TAM family of RTKs to reduce immune suppression, it is noted that deletion of PROS1 in macrophages can also lead to enhanced metastasis, in part through increased production of IL10 that enhances the survival and invasive potential of tumor cells [122].

#### Non-receptor tyrosine kinases

##### Focal adhesion kinase (FAK)

FAK is a multidomain non-receptor tyrosine kinase that classically localizes to focal adhesions where it acts to integrate and transmit signals downstream of specific integrins [123], although non-canonical, transcriptional roles for nuclear FAK have also been reported [124]. Several TKIs with selectivity for FAK have been developed, including PF-0562271, PF04554878/VS-6063/Defactinib, VS-4718, GSK2256098 and IN10018/BI853520 [123]. Increased expression and activation of FAK occurs in a variety of cancers, and FAK contributes in a cancer cell-autonomous fashion to disease progression through effects on cancer cell proliferation, survival and migration. However, FAK also exerts complex effects on the TME that impact upon the efficacy of ICI.

An interesting illustration of this is in PDAC, a malignancy characterized by a desmoplastic stroma that hinders immunosurveillance by restricting access of cytotoxic T cells, as well as high numbers of tumor-associated immune suppressive cells. In an early study, treatment of an orthotopic mouse model of PDAC with the selective FAK inhibitor PF-562271 led to a significant reduction in tumor growth and also a decrease in infiltration of both macrophages and CAFs into the tumor, suggesting that this may relieve the immunosuppression imparted by these cell types [125]. Consistent with these data, pharmacological inhibition or knockdown of FAK in a genetically-engineered mouse model of PDAC led to less tumor fibrosis and reduced infiltration by TAMs and MDSCs [126]. Moreover, treatment with the FAK inhibitor VS-4718 sensitized these tumors to ICI in genetic models, particularly in combination with gemcitabine.

In squamous carcinoma cells, nuclear FAK positively regulates transcription of a cytokine/chemokine gene expression network, including the chemokine CCl5 and cytokine TGFβ, which recruits and expands Tregs and leads to exhaustion of CD8+ T cells. Furthermore, treatment with VS-4718 enabled a CD8+ T cell-mediated anti-tumor response [124]. Subsequent work demonstrated that the T cell co-stimulatory ligand CD80, expressed in a variety of solid and haematological malignancies, represents a potential predictive biomarker for response to anti-FAK therapy. Furthermore, co-targeting FAK and the T cell co-stimulatory receptors OX-40 and 4-1BB represented a potential therapeutic strategy for tumors lacking CD80 expression [127]. Of note, while this work focused on cancer cell intrinsic functions of FAK, this kinase is also expressed by T cells, and inhibition of FAK in this context may sensitize T cells to low affinity tumor antigens and prolong T cell-dendritic cell engagement [128].

This work has led to active clinical trials evaluating the combination of specific FAK inhibitors, particularly Defactinib, with ICI, including pembrolizumab in PDAC, non-small cell lung cancer and mesothelioma (Fig. 6; Table 1).

##### Bruton’s tyrosine kinase (BTK)

BTK is a non-RTK of the TEC family and is best recognized for linking B cell receptor activation to a signalling cascade involving PLCγ2 and NF-κB to promote B cell development and maturation [129]. In addition to its cell-autonomous role in B cell neoplasms, BTK also contributes to the development and progression of solid cancers through its role in specific cell types within the TME, further highlighting its potential as a therapeutic target (Fig. 6; Table 1). One example of this involves CD1d^hi^CD5^+^ regulatory B cells. Treatment of these cells with the BTK TKIs Tirabrutinib or ibrutinib inhibited cell differentiation and production of the immunosuppressive cytokines IL10 and IL35. Likewise, in mice bearing orthotopic KRas^G12D^ pancreatic intra-epithelial neoplastic lesions, Tirabrutinib administration decreased stromal accumulation of regulatory B cells in favour of CD8^+^IFNγ^+^ cytotoxic T cells, and decreased tumor growth [130].

BTK also plays an important role in cells of the myeloid compartment. In MDSCs, BTK is required for their development and function, and targeting of BTK with ibrutinib in mouse models of breast cancer and melanoma resulted in a reduction in MDSC frequency [131]. Furthermore, ibrutinib enhanced the efficacy of anti-PD-L1 therapy against EMT6 murine mammary tumors [131]. Similar results were recently reported in a mouse model of neuroblastoma, where ibrutinib administration increased CD8+ T cell infiltration [132]. Ibrutinib treatment also reprograms MDSCs into mature DCs and enhances MHCII expression, highlighting another mechanism whereby Ibrutinib can enhance the immune response against cancers [133]. Meanwhile in macrophages, BTK promotes the production of pro-inflammatory chemokines and cytokines [129, 134]. Because BTK is expressed in B cells and macrophages, and their crosstalk results in polarization of macrophages to a M2-like tumor-promoting endotype, Ibrutinib treatment of a mouse PDAC model restored T cell-dependent anti-tumor responses and enhanced sensitivity to standard of care gemcitabine [135].

##### Src family kinases (SFKs)

The Src family comprises nine non-receptor tyrosine kinases, including Src, Fyn, Lyn, Lck, Yes and Hck, that regulate diverse cellular processes including cell proliferation, differentiation and migration and contribute in a member-specific manner to development and function of diverse tissues and organs [136]. In addition, aberrant expression and/or activation of particular SFKs is strongly implicated in the progression of many human cancers [137]. Several TKIs in clinical use exhibit some selectivity for SFKs, including Dasatinib, Saracatanib and Bosutinib. However, all of these TKIs also display off-target inhibition of other kinases including the non-RTK Abl [137].

Evidence supporting a role for SFKs in immunosuppression during cancer progression is provided by studies on chronic myeloid leukemia, where Dasatinib treatment led to Treg inhibition, reduced the abundance of MDSCs, and increased both NK cell differentiation and Granzyme B-expressing CD4+ and CD8+ memory T cells [138–140]. Building on these preclinical studies, combining anti-SFK TKIs with anti-PD-1 or PD-L1 ICIs is currently being explored in clinical trials of patients with CML (Fig. 6; Table 1). Furthermore, in syngeneic mouse models of melanoma, sarcoma, colon and breast cancer, Dasatinib administration led to increased CD8+ T cell infiltration, reduced intra-tumoral Treg accumulation, and inhibited tumor growth [141]. However, the relatively broad selectivity profile of dasatinib clouds interpretation of these findings in terms of the kinase(s) responsible. More direct evidence supporting a role for specific SFKs stems from a study in a compound mutant Tgfbr1^KO^/Pten^KO^ mouse model of HNSCC characterized by enhanced expression of Src and Lyn, where Dasatinib treatment reduced tumor size and MDSC recruitment [142]. In this malignancy, Lyn is overexpressed in both the stroma and malignant epithelial cells, and stromal Lyn expression positively correlated with the presence of MDSCs and TAMs [142]. Since administration of an anti-CTLA4 antibody enhanced activation of SFKs in tumors of compound Tgfbr1^KO^;Pten^KO^ mice, the efficacy of combining anti-CTLA4 treatment with Dasatinib [143] was further investigated. This combination synergistically inhibited tumor growth, accompanied by reductions in MDSCs and Tregs, further supporting the concept that SFK targeting may improve the clinical efficacy of ICI. Furthermore, a correlation between Yes and increased presence of Tregs was observed in non-small cell lung cancer [144], and combined treatment with Dasatinib and anti-PD1 resulted in synergistic inhibition of tumor growth, which was accompanied by a reduction in Tregs and was dependent on the presence of CD4+ and CD8+ T cells. The effect of Dasatinib in this study may reflect Yes-dependent reprogramming of the TME, as well as Yes- and Lck-dependent roles in Treg conversion from CD4+ T cells and Treg proliferation [144].

SFKs also play critical roles is macrophages that, according to their maturation stage, express Src, Fyn, Yes, Lyn and the largely myeloid-specific members Hck and Fgr [145]. Hck appears to be the major SFK transducing the CSF-1R-mediated motility signal [145]. Interestingly, Poh et al demonstrated that in mouse models of colon cancer, high Hck activity in TAMs promotes their polarization to a tumor-promoting M2-like endotype and the accumulation of IL6/IL11 family cytokines that promote tumor growth [146]. Furthermore, genetic ablation of Hck or treatment with an Hck-selective small molecule inhibitor reduced tumor growth in mouse models of colon and gastric cancers [146, 147], and when combined with anti-PD1 augmented anti-tumor immune responses [52].

## Conclusions and outlook

Clinical evidence suggests that immune checkpoint blockade is likely to be effective when treatment naïve tumors are already recognized by the immune system, i.e. exhibit a pre-existing CD8+ T cell infiltrate. The lack of spontaneous anti-tumor immune responses may arise from insufficient “visibility” to the immune system, or the tumor’s active subversion of anti-tumor immune responses. The therapeutic challenge in both situations is to sufficiently push tumors along the “immune-incline” to elicit a response that results in clinical remission while limiting collateral immune damage. Clinical observations suggest that specific tumors will show greatest susceptibilities to only some of the therapeutic approaches available to increase tumor immunogenicity. These treatments include cancer vaccines, oncolytic virus, co-stimulatory molecules, radiation/chemotherapy, adoptive cell therapy among other modalities. Likewise, understanding and exploiting the learnings from the immune cycle provides a multitude of opportunities to overcome myeloid cell polarisation, aberrant angiogenesis, excessive chemokine/cytokine expression and other immunosuppressive mechanisms in the TME. Individually or collectively these mechanisms are likely to yield therapeutic vulnerabilities that can be exploited against a backbone of overcoming local immune suppression by inhibition of PD-1/PD-L1 interaction. A deeper molecular understanding of the immune cycle will also allow us to rationalise approaches other than concomitant initiation of combination therapies and to exploit opportunities that arise from sequential therapies and priming with a short exposure to one therapy before switching to or combining with another therapy [148, 149]. Indeed, preclinical studies suggest that sequencing individual treatments may also create synergies by raising the threshold for the development of resistance. Finally, the development of novel biomarkers and companion diagnostics other than the current focus on TMB and PD-1 expression [150], will provide a better understanding on how to combine ICI most effectively with specific tyrosine kinase inhibitors and other targeted treatment modalities.

## Data Availability

Not applicable.
